# Supplementary material for: Weakened AMOC related to cooling and atmospheric circulation shifts in the last interglacial Eastern Mediterranean
Source: Nat Commun. 2023 Aug 25;14:5180. doi: 10.1038/s41467-023-40880-z (PMC10449873; doi:10.1038/s41467-023-40880-z)
Supplement: Supplementary file 1 — Supplementary Information [file 41467_2023_40880_MOESM1_ESM.pdf]

Supplementary Information for

Weakened AMOC related to cooling and atmospheric circulation shifts in the last interglacial Eastern Mediterranean

Levy et al.

Peqi'in Cave temperatures derived using the $\Delta\delta^{18}O$ method.

The LIG calcite crystallisation temperatures at Peqi'in Cave were reconstructed using the $\Delta\delta^{18}O$ calcite-fluid inclusion water method by applying (eq. 1):

$$10^3\, ln(^{18}\alpha_{CaCO_3/H_2O}) = A \times 10^3/T - B \quad (1)$$

Where $^{18}\alpha$ is the fractionation between calcite and water, T is in kelvin, and A and B parameters equal to 17.57 and 29.13, respectively, after ref.[1], or 16.1 and 24.6, respectively, after ref.[2]. In the study by ref.[1] the A and B parameters were calculated using calcites formed at slow crystallization rates from Devils Hole and Laghetto Basso (Corchia Cave). Comparatively, in the study by ref.[2] parameters were calculated using compiled measured data across a range of temperatures and cave environments.

At Peqi'in Cave the LIG $\Delta\delta^{18}O$ temperatures exhibit high variability but are generally equal to or lower than respective GDGT-derived TEX_{86} temperatures (Supplementary Fig. 1A; Supplementary Data). The $\Delta\delta^{18}O$ temperatures calculated using A and B parameters by ref.[1] (Supplementary Fig. 1A - blue) conform better to the TEX_{86} temperatures (Supplementary Fig. 1A -green) than temperatures calculated using the parameters by ref.[2] (Supplementary Fig. 1A - red). In both cases, $\Delta\delta^{18}O$ temperatures reveal that a part of the early-LIG (*ca.* 127 ka) is several degrees cooler than TEX_{86}. Although these low temperatures are adequate for allowing speleogenesis, given that the early-LIG was marked by a thermal optimum suggests that these cooler early-LIG $\Delta\delta^{18}O$ temperatures are anomalous. Analytical constraints during measurement such as the evaporation of fluid inclusion water from samples prior to crushing or calcite decrepitation would result in ^{18}O-enriched fluid inclusion $\delta^{18}O$ and resulting calculated temperatures that are too high[3]. However, here the early-LIG temperatures are cooler than

expected which may suggest that either the fluid inclusion $\delta^{18}O$ are ^{18}O-depleted relative to the accompanying $\delta^{18}O_{calcite}$ or that the $\delta^{18}O_{calcite}$ is ^{18}O-enriched relative to fluid inclusion water $\delta^{18}O$. Mechanisms which could have resulted in these low early-LIG temperatures include: 1) wintertime speleothem formation (i.e., the $\Delta\delta^{18}O$ temperatures reflect winter temperatures). Indeed, during the early-LIG there was high seasonality[4] mainly as a result of amplified seasonal insolation differences[5] (precession minima; Supplementary Fig. 1B, 1C, 1D). This may have resulted in cooler wintertime surface temperatures at Peqi'in. However, it is unlikely that temperatures within Peqi'in Cave would have fluctuated significantly between seasons; 2) decoupling between fluid inclusion water $\delta^{18}O$ and $\delta^{18}O_{calcite}$ due to seasonal bias affecting the fluid inclusion distribution/fabric (i.e., as suggested for a speleothem from Jiangjun Cave, China[6]). A speleothem dating to the high-precession period of the LIG at the nearby Soreq Cave reveals strong seasonality imprinted on the $\delta^{18}O_{calcite}$ which may argue in favour of this mechanism[7]; 3) kinetic isotope fractionation which affected the $\delta^{18}O_{calcite}$ but not the fluid inclusion $\delta^{18}O$[8]. For example, prior calcite precipitation (PCP), where calcite precipitation occurs before the cave drip water enters through the cave[9]. PCP leads to ^{18}O-enrichment of the dissolved HCO_3^-. If equilibrium between HCO_{3-} and H_2O is not re-established following PCP the precipitated $\delta^{18}O_{calcite}$ will increase and shift from O isotope equilibrium with the corresponding drip water. The fact that an early-LIG $\delta^{18}O_{calcite}$ minimum at Peqi'in is missing unlike in other speleothem $\delta^{18}O_{calcite}$ records (i.e. Soreq, Corchia) and marine planktic archives (i.e. *G. ruber* record from LC21; Fig. 3C) is evidence which may support this mechanism. Given that the early-LIG $\delta^{18}O_{calcite}$ at Peqi'in is higher than that at Soreq Cave is also a noteworthy observation in support of this mechanism; 4) diagenetic alteration of speleothem calcite. During diagenetic recrystallization of $CaCO_3$ the $\delta^{18}O_{calcite}$ and fluid inclusion water δ^2H is typically unaffected[8].

Fluid inclusion $\delta^{18}O$ is more susceptible to alteration compared to $\delta^{18}O_{calcite}$ due to low water yield volumes. Warm cave environments may promote formation of well crystallized calcite, whereas carbonates formed in colder caves may be more susceptible to isotopic alteration[8]. In a region where meteoric rainfall is close to GMWL values (*d-excess* ~ 10‰) one may be able detect this process given a shift towards anomalously high fluid inclusion *d-excess* values. However, for this particular region, the Peqi'in Cave LIG fluid inclusion water *d-excess* maxima, together with accompanying $\delta^{18}O$ and $\delta^{2}H$, cannot be assumed as anomalous for neither the LIG[3], modern moisture[10] or modern rainfall[11]. Additionally, there is indication that fluid inclusion $\delta^{2}H$ slightly shifts towards more negative values during the $\delta^{18}O$ negative excursion during the early-LIG which argues that calcite recrystallization is unlikely.

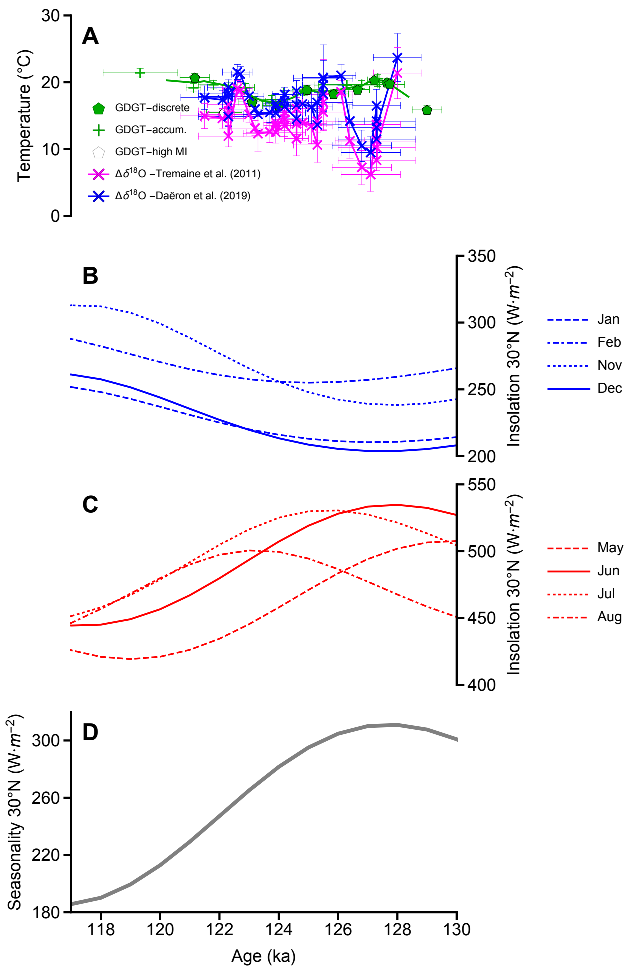

Supplementary Fig. 1: Peqi'in temperatures, insolation records and seasonality. (**A**) TEX_{86} temperatures: discrete samples (green pentagons), accumulated (green crosses) and high methane index sample (white pentagon). $\Delta\delta^{18}O$ temperatures using parameters from ref.[1] (blue crosses) and ref.[2] (purple crosses) methods. TEX_{86} temperature error bars were estimated based on repeated measurements of a speleothem standard and, for accumulated samples, error propagation. The $\Delta\delta^{18}O$ temperature error was calculated from error propagation of the analytical and standard replicate uncertainties of fluid inclusion and calcite O isotopes. (**B**) Winter month mean insolation at 30°N[5] (blue dashed and regular lines). (**C**) Summer month mean insolation at 30°N[5] (red dashed and regular lines) (**D**) Seasonality (MJJ –NDJ insolation; grey line).

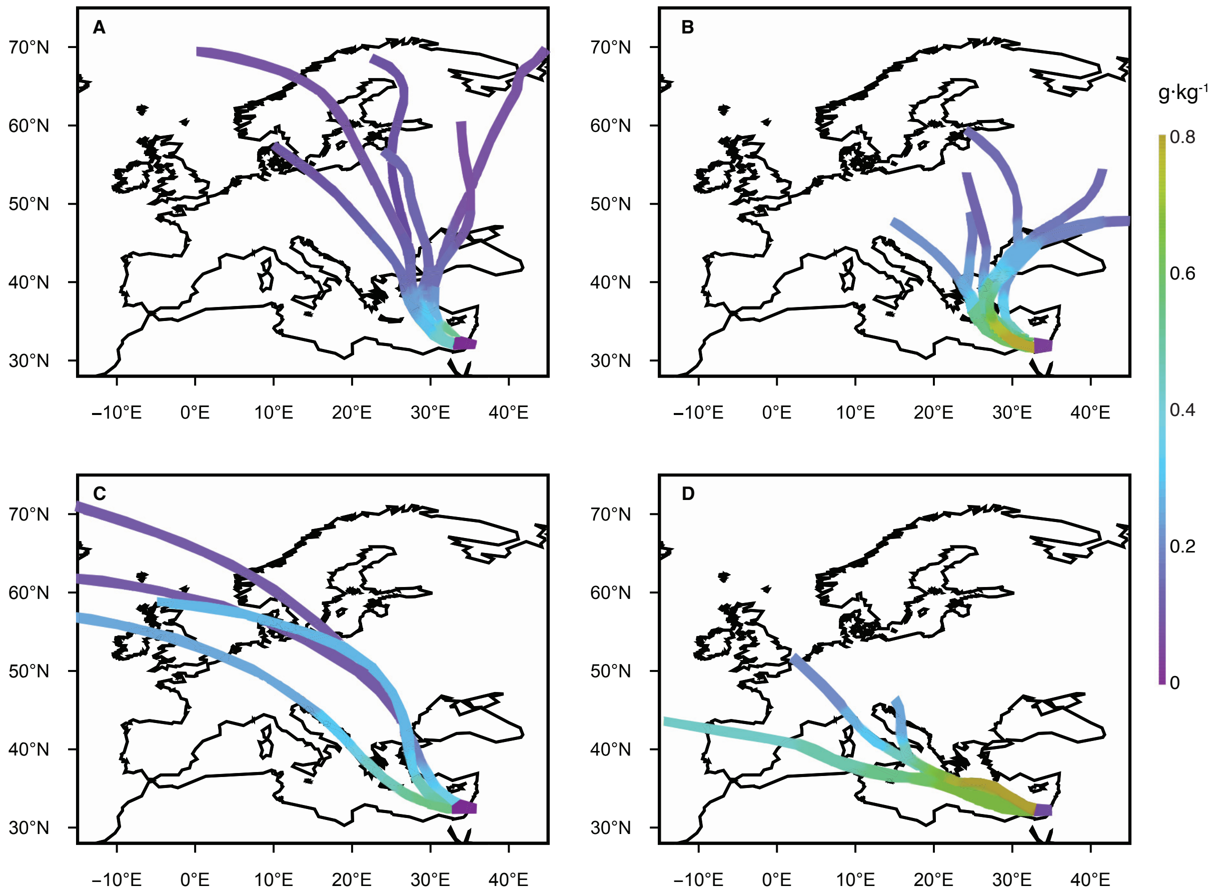

Supplementary Fig. 2: Mean backward trajectories of clusters for >15 mm rainfall events between 1995-2021 at Soreq Cave. (**A**) N-cluster (Cluster #0). (**B**) NW(short) cluster (Cluster #1). (**C**) NW-cluster (Cluster #2). (**D**) W-cluster (Cluster #3). The colour along the trajectories marks specific humidity, such that colour changes illustrate the moisture uptake position.

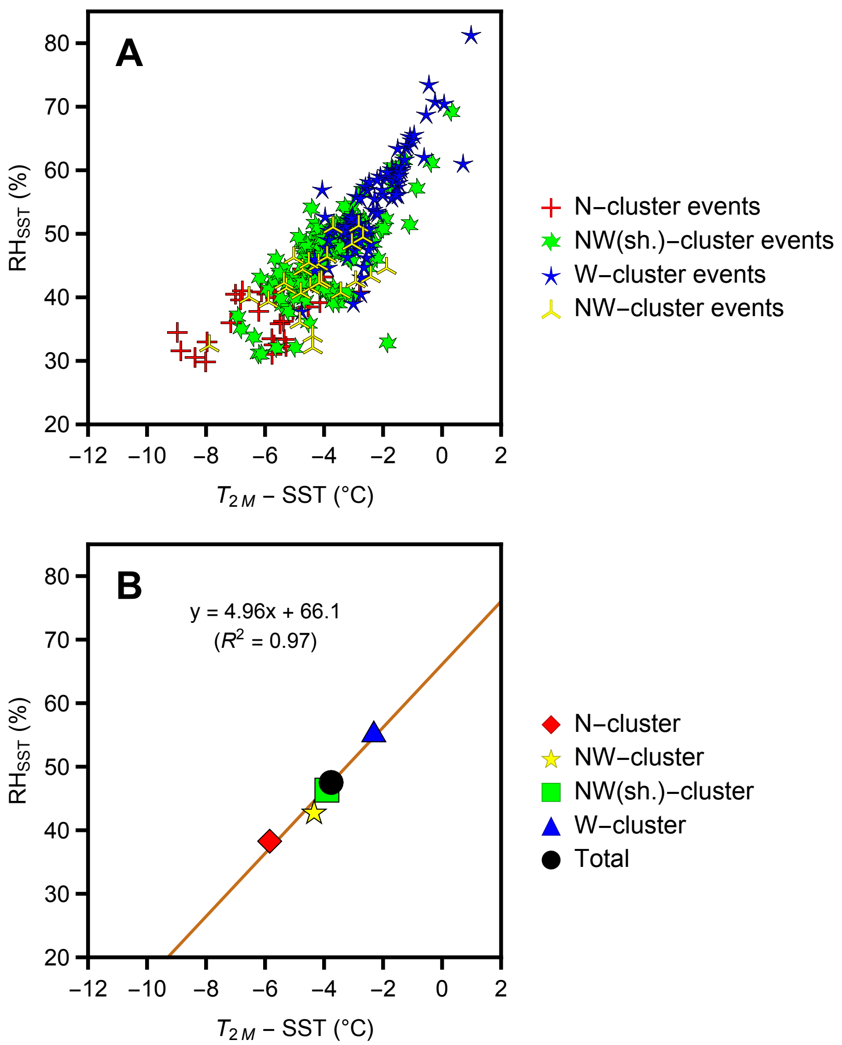

Supplementary Fig. 3. Moisture uptake relative humidity (RH_{SST}) vs. near surface air temperature (T_{2M}) minus sea surface temperature (SST). (**A**) Individual rainfall events >15 mm at Soreq (coloured according to the respective dominant cluster; N-cluster= red cross; NW(short)=green six-pointed star; W-cluster=blue five-pointed star; NW-cluster=yellow inverted Y markers). (**B**) Accumulated rainfall of clusters (red diamond= N-cluster, yellow star = NW.-cluster; green square=NW(short)-cluster; blue triangle= W-cluster) and total accumulated rainfall (black disk). The linear regression and equation calculated for this data are shown (orange line).

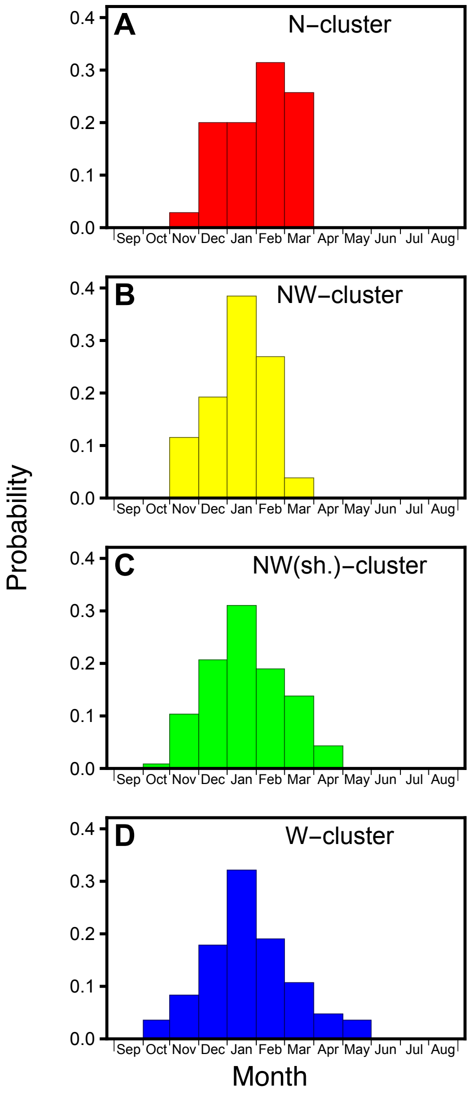

Supplementary Fig. 4: Probability histogram plots of cluster frequency for >15mm rainfall events. (**A**) N-cluster (red). (**B**) NW-cluster (yellow). (**C**) NW(sh.)-cluster (green). (**D**) W-cluster (blue). A normal distribution of rainfall centred around January for the W-cluster and NW(sh.)-cluster is evident. The rainfall season length differs between clusters, with rainfall from the N-cluster occurring between November and March and rainfall from the W-cluster from October until May.

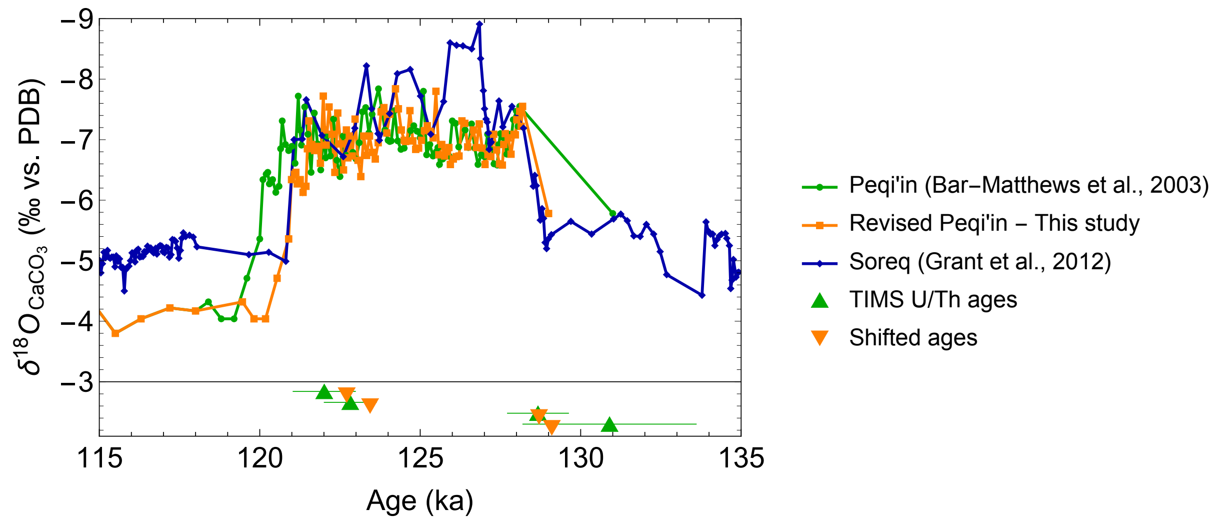

Supplementary Fig. 5: Peqi'in Cave $\delta^{18}O_{calcite}$ record with revised chronology. The Soreq Cave $\delta^{18}O_{calcite}$ record[12,13] (blue line) with Peqi'in Cave original[12] (green line) and revised (shifted within 2σ; orange line) $\delta^{18}O_{calcite}$ records. Lower pane shows TIMS derived U/Th ages and respective ±2σ (green triangles) for PEK-9[12] and post-shifted anchor positions for revised Peqi'in LIG record chronology (orange inverted triangles).

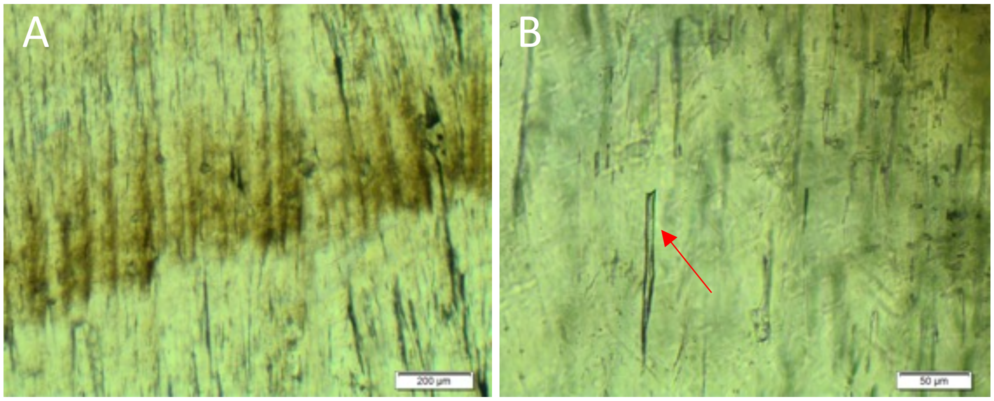

Supplementary Fig. 6: Thin section images of PEK-9. (**A**) Image illustrating axial parallel fluid inclusions and (**B**) thorn shaped fluid inclusion.

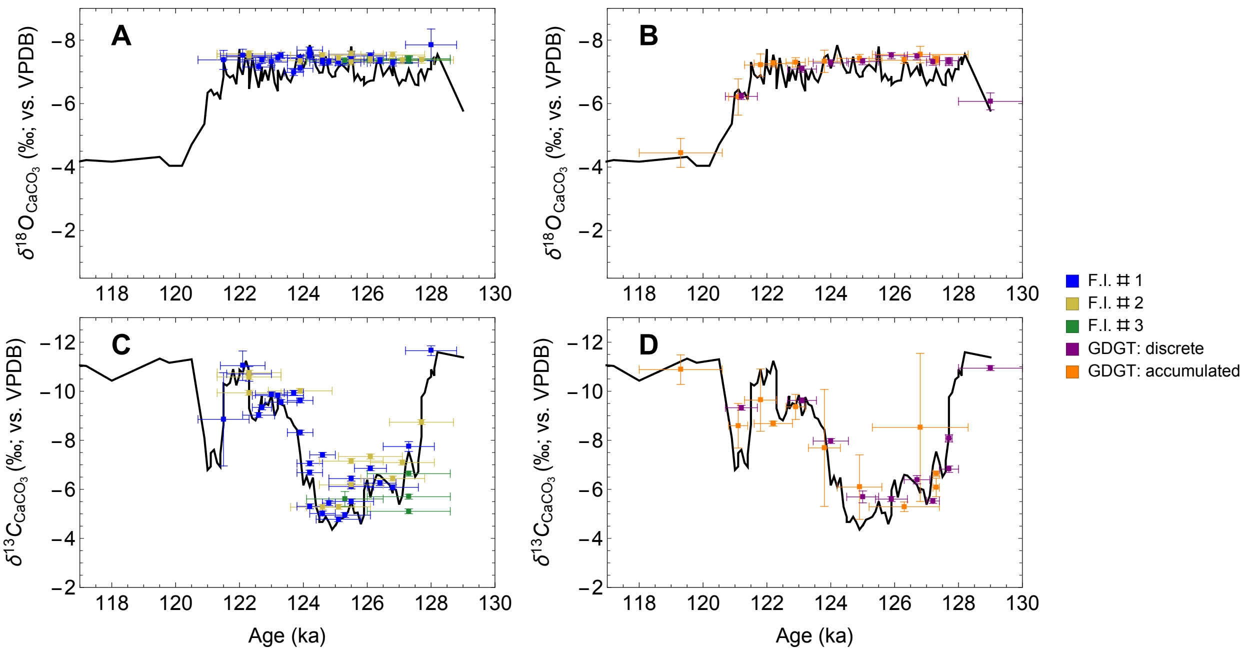

Supplementary Fig. 7: Fluid inclusion and TEX$_{86}$ sample $\delta^{18}O_{calcite}$ and $\delta^{13}C_{calcite}$. (**A**) Fluid inclusion sample $\delta^{18}O_{calcite}$ (markers coloured according to batch: #1= blue; #2= yellow; #3= green). (**B**) TEX$_{86}$ sample $\delta^{18}O_{calcite}$ (discrete= purple; accumulated= orange). (**C**) Fluid inclusion sample $\delta^{13}C_{calcite}$. (**D**) TEX$_{86}$ sample $\delta^{13}C_{calcite}$. Additionally, the Peqi'in Cave $\delta^{18}O_{calcite}$ (black lines in A and B) and $\delta^{13}C_{calcite}$ (black lines in C and D) timeseries records are shown.

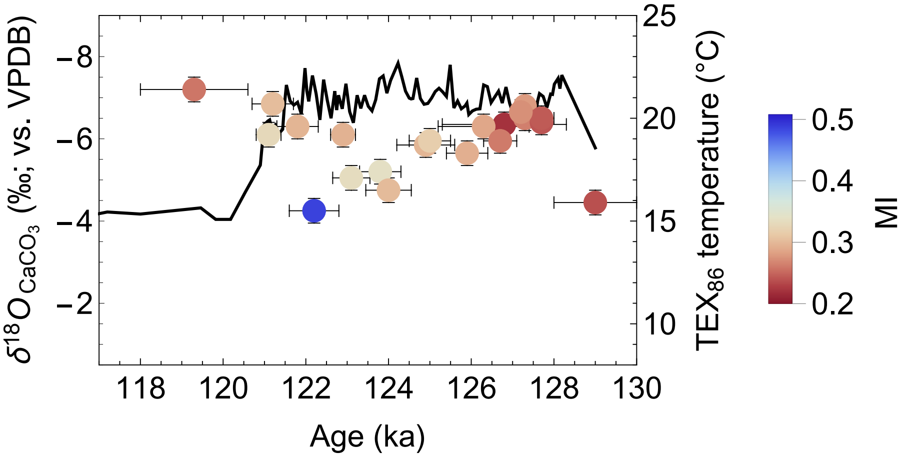

Supplementary Fig. 8. Peqi'in Cave TEX_{86} results and MI. Isoprenoid GDGT derived TEX_{86} temperatures (disks) coloured according to the respective Methane Index (MI). The samples have a MI between 0.22 to 0.34 apart from one sample with high MI value of 0.49 (blue disk). None of the samples exceed the GDGT-0 / Crenarchaeol threshold >2. Additionally the Peqi'in Cave record $\delta^{18}O_{calcite}$ (black line) is shown.

Supplementary Table 1: Details of ages for PEK-9 for the laminae relevant in this study after ref.[12].

Laminae #	Age (ka)	2σ
D2	130.9	2.7
D1	128.67	0.95
C	122.82	0.81
B	122.01	0.97

Supplementary Table 2: Rainfall collection site names, respective group and site coordinates after ref.[11,14,15].

Site	Group	Coordinates
Hermon, Lower Cable	North	33.308° N, 35.772° E
Neve Ativ	North	33.262° N, 35.740° E
Peqi'in	North	32.976° N, 35.329° E
Mikhmanim	North	32.906° N, 35.326° E
Haifa 1 (Port)	North	32.817° N, 35.010° E
Haifa 2 (Tel Shikmona)	North	32.826° N, 34.957° E
Beit Dagan	Central	31.995° N, 34.822° E
Soreq	Central	31.756° N, 35.023° E
Jerusalem, Arnona	Central	31.748° N, 35.220° E
Jerusalem (Geological Survey)	Central	31.775° N, 35.194° E
Beer Sheva	Central	31.278° N, 34.823° E

Supplementary References

1. Daëron, M. et al. Most Earth-surface calcites precipitate out of isotopic equilibrium. *Nat. Commun.*, **10**, p.429 (2019).

2. Tremaine, D. M., Froelich, P. M. & Wang, Y. Speleothem calcite farmed in situ: Modern calibration of δ18O and δ13C paleoclimate proxies in a continuously-monitored natural cave system. *Geochim. Cosmochim. Ac.* **75**, 4929-4950 (2011).

3. Matthews, A., Affek, H. P., Ayalon, A., Vonhof, H. B. & Bar-Matthews, M. Eastern Mediterranean climate change deduced from the Soreq Cave fluid inclusion stable isotopes and carbonate clumped isotopes record of the last 160 ka. *Quat. Sci. Rev.* **272**, 107223 (2021).

4. Felis, T. et al. Increased seasonality in Middle East temperatures during the last interglacial period. *Nature* **429**, 164-168 (2004).

5. Laskar, J. et al. A long-term numerical solution for the insolation quantities of the Earth. *Astron. Astrophys.* ***428***, 261-285 (2004).

6. Wassenburg, J. A. et al. Penultimate deglaciation Asian monsoon response to North Atlantic circulation collapse. *Nat. Geosci.* **14**, 937-941 (2021).

7. Orland, I. J. et al. Resolving seasonal rainfall changes in the Middle East during the last interglacial period. *Proc. Natl. Acad. Sci.* **116**, 24985-24990 (2019).

8. Demény, A. et al. Recrystallization-induced oxygen isotope changes in inclusion-hosted water of speleothems–Paleoclimatological implications. *Quat. Int.*, ***415***, 25-32 (2016).

9. Deininger, M. et al. Are oxygen isotope fractionation factors between calcite and water derived from speleothems systematically biased due to prior calcite precipitation (PCP)?. *Geochim. Cosmochim. Ac.*, ***305***, 212-227 (2021).

10. Gat, J. R. & Carmi, I. Effect of climate changes on the precipitation patterns and isotopic composition of water in a climate transition zone: case of the eastern Mediterranean Sea area. *IAHS Spec. Publ.* **168**, 513-523 (1987).

11. Ayalon, A., Bar-Matthews, M. & Schilman, B. *Rainfall isotopic characteristics at various sites in Israel and the relationships with unsaturated zone water* (GSI/16/04, Geological Survey of Israel, 2004).

12. Bar-Matthews, M., Ayalon, A., Gilmour, M., Matthews, A. & Hawkesworth, C. J. Sea–land oxygen isotopic relationships from planktonic foraminifera and speleothems in the Eastern Mediterranean region and their implication for paleorainfall during interglacial intervals. *Geochim. Cosmochim. Ac.* **67**, 3181-3199 (2003).

13. Grant, K. M. et al. Rapid coupling between ice volume and polar temperature over the past 150,000 years. *Nature* **491**, 744–747 (2012).

14. Goldsmith, Y. et al. The modern and Last Glacial Maximum hydrological cycles of the Eastern Mediterranean and the Levant from a water isotope perspective. *Earth Plan. Sci. Lett.* **457**, 302-312 (2017).

15. Ayalon, A., Bar-Matthews, M. & Sass, E. Rainfall-recharge relationships within a karstic terrain in the Eastern Mediterranean semi-arid region, Israel: δ18O and δD characteristics. *J. Hydrol.* **207**, 18-31 (1998).
