# Supplementary material for: Weakened AMOC related to cooling and atmospheric circulation shifts in the last interglacial Eastern Mediterranean
Source: Nat Commun. 2023 Aug 25;14:5180. doi: 10.1038/s41467-023-40880-z (PMC10449873; doi:10.1038/s41467-023-40880-z)
Supplement: Supplementary file 3 — Description of Additional Supplementary Files [file 41467_2023_40880_MOESM3_ESM.pdf]

Description of Additional Supplementary Files

Supplementary Data 1:

This file contains speleothem related data namely: calcite, fluid inclusion and TEX_{86} temperature records. It also includes data for modern rainfall stable isotopes, clusters, accumulated rainfall and Mediterranean Oscillation Index.

Supplementary Code: The Supplementary Code is for calculating backward trajectory moisture uptake conditions and clusters.
